# Effects of the Marinating Process on the Quality Characteristics and Bacterial Community of Leisure Dried Tofu

**DOI:** 10.3390/foods12040841

**Published:** 2023-02-16

**Authors:** Tao Wu, Zhanrui Huang, Liangzhong Zhao, Xiaohu Zhou, Hao Chen, Xiaojie Zhou, Ming Li, Jinsong Zhou, Yingyi Lin

**Affiliations:** 1 Hunan Provincial Key Laboratory of Soybean Products Processing and Safety Control, College of Food and Chemical Engineering, Shaoyang University, Shaoyang 422000, China; 2 Jinzai Food Group Co., Ltd., Yueyang 414022, China; 3 Beijing Kangdeli Machinery Manufacturing Co., Ltd., Beijing 100074, China

**Keywords:** leisure dried tofu, marinade, marinating process, quality characteristics, bacterial community

## Abstract

Leisure dried tofu (LD-tofu) was prepared using two different marinating processes: the repeated heating method (RHM) and the vacuum pulse method (VPM). The quality characteristics and bacterial community succession of LD-tofu and the marinade were evaluated. The results showed that the nutrients in LD-tofu were easily dissolved into the marinade during the marinating process, while the protein and moisture content of RHM LD-tofu changed most dramatically. With the increase in marinade recycling times, the springiness, chewiness and hardness of VPM LD-tofu increased significantly. The total viable count (TVC) of the VPM LD-tofu decreased from the initial value of 4.41 lg cfu/g to 2.51–2.67 lg cfu/g as a result of the marinating process, which had a significant inhibitory effect. Additionally, 26, 167 and 356 communities in the LD-tofu and marinade were detected at the phylum, family and genus levels, respectively. Pearson correlation analysis showed that *Pseudomonadaceae*, *Thermaceae* and *Lactobacillaceae* were closely related to the quality characteristics of LD-tofu, whereas *Caulobacteriaceae*, *Bacillaceae* and *Enterobacteriae* were closely related to the marinade. The present work provides a theoretical basis for the screening of functional strains and quality control in LD-tofu and marinade.

## 1. Introduction

Leisure dried tofu (LD-tofu) is a reprocessed soybean product made from tofu through drying, marinating, sterilisation and other processes [1,2,3]. The LD-tofu retains the original nutritional composition of tofu and is popular with consumers due to its unique flavour. Hence, LD-tofu has become a typical representative of leisure food, which has promoted the rapid expansion of the LD-tofu market [1,4].

The quality characteristics of LD-tofu are the core element of its grading, and monitoring the changes in LD-tofu quality is one of the key aspects for the healthy growth of the industry [1,4]. The marinating process involves the repeated heating of the food in the pre-cooked marinade (cooked from spices, rapeseed oil, pig bones and other materials), which is the critical control point affecting the quality of LD-tofu [3]. The texture and quality of tofu and its products could change as a result of nonstandard processes or inaccurate parameters, leading to serious financial losses [5,6,7]. In order to reduce production costs, producers often use deteriorated raw materials or recycled marinade (even hundreds of times) to make LD-tofu, which can readily cause microbial contamination [8,9]. Given the drawbacks of traditional marinating processes, our team developed a vacuum pulse marinating process and supporting equipment, which could shorten the marinating time and improve the flavour as well as colour of LD-tofu [1]. However, the changes in the quality and microbial diversity of the LD-tofu during this marinating process are unclear.

Controlling the microbial community is a key factor in food quality and safety control [10,11]. The complex process in tofu and tofu product production often utilizes fermented soybean whey as a coagulant, resulting in complex and diverse microbial communities [12,13]. In addition, LD-tofu has rich nutrition and high water activity, which is suitable for the reproduction and metabolism of microorganisms [4]. Even though the microbial characteristics and specific spoilage microorganisms of fresh soybean products have been studied [14,15], it is still unclear how the bacterial communities in LD-tofu and marinade progress under marinade recycling. Therefore, clarifying the correlation between the succession of bacterial communities and the quality changes in LD-tofu during the marinating process, as well as identifying the key bacterial communities, is a new strategy to avoid quality deterioration and achieve safety control in LD-tofu production.

To evaluate the effect of the marinating process on the quality characteristics and bacterial community of LD-tofu, LD-tofu was prepared by using repeatedly recycled marinade (0, 30, 60, 90, 120, 150 cycles) via the repeated heating method (RHM) and vacuum pulse method (VPM) in this study. The changes in the nutritional composition, texture characteristics and total viable count (TVC) of the LD-tofu and marinade were determined by conventional physicochemical methods and a texture analyser. The bacterial community succession in the LD-tofu and marinade was analysed by high-throughput sequencing. The correlation between the target microorganisms and the quality characteristics was determined. The purpose of this work was to provide a theoretical basis for the sterilisation and quality control of LD-tofu while supporting the safe and efficient production of marinated leisure food.

## 2. Materials and Methods

### 2.1. Sample Preparation and Collection

**Figure 1:**
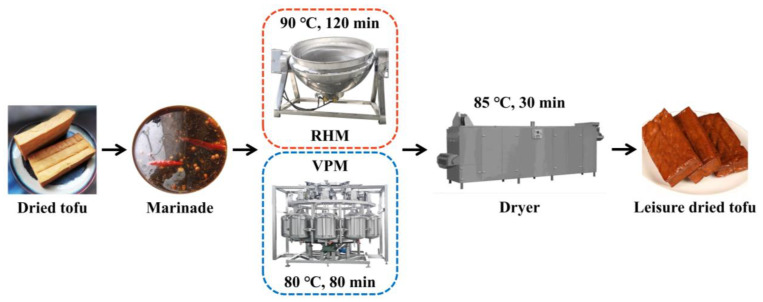
Marinating process flow chart of LD-tofu by RHM and VPM.

The LD-tofu (8 × 3 × 1.5 cm, 25.00 ± 0.50 g) were obtained from different marinating processes according to Figure 1 [1]. Six groups—0 (control), 30, 60, 90, 120 and 150—were created according to the number of marinade recycling cycles. To produce LD-tofu, dried tofu (10 pieces/time) was marinated by RHM (90 °C, 120 min) in a cooking pot and VPM (80 °C, 80 min; vacuum: 0.03 MPa; no. of pulses: 3) using vacuum pulse equipment (Kangdeli Machinery Equipment Manufacturing Co., Ltd., China). The ratio of dried tofu and marinade was 1:40 (g/mL). A marinade sample (100 mL) was taken from each group after the marinating process to determine the nutritional components, textural properties and TVC. The fresh marinade was added for the next marinating process according to the volume. LD-tofu was dried in a dryer at 85 °C for 30 min and after cooling the samples were collected for analysis. Additionally, LD-tofu and marinade samples were analysed for bacterial community composition during the early (50), middle (100) and late (150) stages.

### 2.2. Chemical Analysis

The moisture, protein and fat contents of LD-tofu were determined by using direct drying method, Kjeldahl method and ether extraction method, respectively. Additionally, the protein conversion factor was 6.25 [16].

### 2.3. Textural Properties

The hardness, chewiness and springiness of LD-tofu were measured at four corners and the centre of the LD-tofu using a Texture Analyzer (LS-5, AMETEK, Berwyn, PA, USA) [17]. The following are the main parameters: P/0.5 cylindrical flat bottom probe; triggering force of 0.05 N; sample flat surface height of 20 mm; test front, middle and final speeds of 2.0 mm/s, 1.0 mm/s and 1.0 mm/s; acquisition rate of 50.0 PPS (pulses per second).

### 2.4. Total Viable Counts

Five grams of LD-tofu were homogenized with sterilized saline solution (0.85%, 45 mL) for 3 min (10,000 r/min). All homogenates were diluted ten-fold serially using saline (0.85%). Then, the diluted sample (0.1 mL) was spread onto plate count agar (Beijing Aoboxing Bio-tech Co., Ltd., Beijing, China) and incubated at 37 °C for 48 h to determine the TVC [18].

### 2.5. DNA Extraction and PCR Amplification

Microbial DNA was extracted using the HiPure Soil DNA Kit (Magen, Guangzhou, China) according to the manufacturer’s protocols [19]. The 16S rDNA V3–V4 region of the ribosomal RNA was amplified by PCR (95 °C for 2 min, followed by 27 cycles at 98 °C for 10 s, 62 °C for 30 s and 68 °C for 30 s, then a final extension at 68 °C for 10 min) using primers 341F: CCTACGGGNGGCWGCAG and 806R: GGACTACHVGGGTATCTAAT, where the barcode was an eight-base sequence unique to each sample. The PCR reactions were performed in triplicate using a 50 μL mixture containing KOD Buffer (10×, 5 μL), dNTPs (2.5 mM, 5 μL), primers (5 μM, 1.5 μL of each), KOD polymerase (1 μL) and template DNA (100 ng).

### 2.6. Illumina HiSeq Sequencing

Amplicons were extracted from 2% agarose gel and purified using the AxyPrep DNA Gel Extraction Kit (Axygen Biosciences, Union City, CA, USA) according to the manufacturer’s instructions and quantified using the ABI StepOnePlus Real-Time PCR System (Life Technologies, Foster City, CA, USA). Purified amplicons were pooled equimolarly and paired-end sequenced (2 × 250 bp) on an Illumina platform according to the standard protocols [19].

### 2.7. Analysis of Bacterial Community Structure

To obtain high-quality clean reads, raw reads were filtered according to the following rules using FASTP (https://github.com/OpenGene/fastp; accessed on 24 March 2021). Then, paired-end clean reads were merged as raw tags using FLSAH (version 1.2.11) with a minimum overlap of 10 bp and a mismatch error rate of 2%. Noisy sequences of raw tags were filtered by the QIIME (version 1.9.1) pipeline to obtain high-quality clean tags. All chimeric tags were removed to obtain effective tags for further analysis. The effective tags were clustered into operational taxonomic units (OTUs) with ≥97% similarity using the UPARSE pipeline. The tag sequence with the highest abundance was selected as the representative sequence within each cluster. The α-diversity indexes (Shannon, Simpson, Chao1 and ACE) and β-diversity index (principal component analysis (PCA)) were calculated by QIIME.

### 2.8. Statistical Analysis

Each measurement was repeated three times, and the results were expressed as the mean ± standard deviation (SD). SPSS 22.0 software (IBM, Chicago, IL, USA) and Origin 9.1 software (OriginLab, Northampton, MA, USA) were used for data analysis. ANOVA and Duncan’s multiple range test (*p* < 0.05) were used to determine the statistical difference between the groups.

## 3. Results and Discussion

### 3.1. Effects of Different Marinating Processes and Different Marinade Recycling Cycles on the Basic Nutrients in LD-tofu

**Figure 2:**
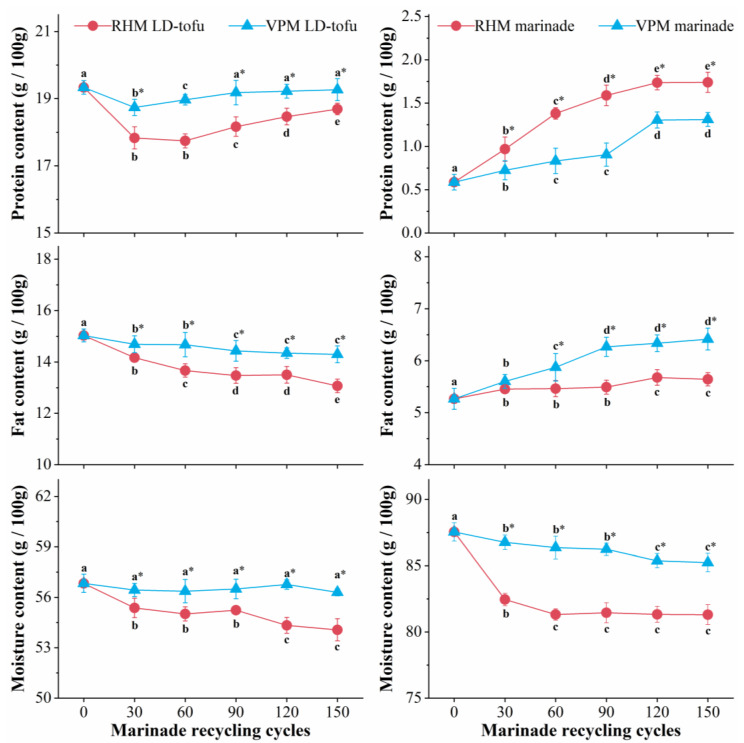
Effects of marinating method and marinade recycling cycles on the basic nutrients of LD-tofu and marinade. Superscripts with different letters in a row are significantly different between groups (*p* < 0.05). * indicates a significant difference between RHM groups and VPM groups (*p* < 0.05).

The changes in the basic nutrients of the LD-tofu and marinade under different marinating processes and different marinade recycling cycles are shown in Figure 2. With the increase in marinade recycling cycles, the protein content of the RHM LD-tofu and VPM LD-tofu decreased at 0–30 recycling cycles and increased at 30–150 recycling cycles. Moreover, the protein content of the RHM LD-tofu changed more significantly than that of VPM LD-tofu. The fat content of the RHM LD-tofu and VPM LD-tofu decreased significantly with the decline in marinade recycling cycles (*p* < 0.05), from 15.03 g/100 g to 13.07 g/100 g and 14.30 g/100 g, respectively. With the increase in marinade recycling cycles (*p* < 0.05), the moisture content of the VPM LD-tofu changed marginally, while the moisture content of the RHM LD-tofu significantly decreased, reaching 54.07 g/100 g after 150 recycling cycles. The decrease in LD-tofu fat and protein content could be attributed to the fact that the soluble protein and fatty acid in the LD-tofu gradually dissolved or hydrolysed during the marinating process. Another reason could be that the protein oxidative denaturation was induced by peroxide and free radicals produced by the lipid oxidation of the LD-tofu under the high-temperature marinating process [20,21]. The LD-tofu is a highly gelatinous product that forms by protein–protein and protein–water interactions [22,23]. Therefore, the oxidative denaturation of LD-tofu protein is bound to destroy the protein gel structure, resulting in a reduction in its water-holding capacity (WHC) and water content [24]. Due to the higher temperature of the RHM process and the open environment, the moisture content of the RHM LD-tofu decreased significantly compared with the VPM groups. The above results also confirmed that there was a direct relationship between lipid oxidation, protein oxidation and the WHC [25,26].

With the increase in marinade recycling cycles, the moisture content of the marinade in the VPM groups decreased marginally and significantly decreased (*p* < 0.05) in the RHM groups from the initial value of 87.54 g/100 g to 81.31 g/100 g. This was mainly due to the evaporation of water caused by the open, high-temperature marinating process. With the increase in marinade recycling cycles, the marinade protein and fat content in the RHM and VPM groups significantly increased (*p* < 0.05). At 150 recycling cycles, the protein content of VPM marinade and RHM marinade was 1.74 g/100 g and 1.31 g/100 g, while the fat content was 6.42 g/100 g and 5.64 g/100 g, respectively. This could be attributed to the dissolution of nutrients from the LD-tofu and the reduction in the moisture content of the marinade. It is interesting to note that while the protein and moisture content of the marinade in the RHM groups increased more than in the VPM groups, the fat content increased less than in the VPM groups. This phenomenon may have been caused primarily by the difference in marinating temperatures between the RHM and VPM, which resulted in the difference in the degree of water evaporation and fat oxidation in the marinade. Additionally, the low-fat content of the marinade in the RHM groups was attributed to the floating oil droplets, which were produced in the upper layer of the marinade under VPM processing and were required to be removed before the initiation of a new marinating process.

### 3.2. Effects of Different Marinating Processes and Different Marinade Recycling Cycles on the Textural Characteristics of LD-tofu

Hardness, chewiness and springiness are the most commonly used indicators to evaluate the textural characteristics of LD-tofu. Hardness refers to the force required to deform the food to a certain extent, chewiness refers to the energy required to chew the solid sample for swallowing, and springiness refers to the ability of the food to quickly recover its original shape following the application of a force [22,27]. The springiness, chewiness and hardness of the LD-tofu in the RHM and VPM groups were measured by a texture analyser in this study (Table 1). With the increase in the marinade recycling cycles, the hardness and chewiness of the LD-tofu in the RHM and VPM groups first marginally decreased and then significantly increased. Additionally, the hardness and chewiness of the LD-tofu in the VPM groups were significantly higher than in the RHM groups (*p* < 0.05). The changes in the hardness and chewiness of the LD-tofu were consistent with the changes in protein content but opposite to those in water content. These results indicated that the protein and water contents of the LD-tofu were closely related to hardness and chewiness, which was consistent with the results of Bu et al. [4]. This was primarily attributed to the denaturation of LD-tofu protein and the decline in water content, which ultimately increased the density of the protein gel and enhanced the hardness as well as chewiness of the LD-tofu.

Additionally, with the increase in marinade recycling cycles, the springiness of the RHM LD-tofu and VPM LD-tofu increased marginally. High-quality LD-tofu has good springiness, a good taste and soft texture [4,28]. These results showed that the VPM LD-tofu had better gel properties and textural characteristics than the RHM LD-tofu.

### 3.3. Effects of Different Marinating Processes and Different Marinade Recycling Cycles on the TVC of LD-tofu

**Figure 3:**
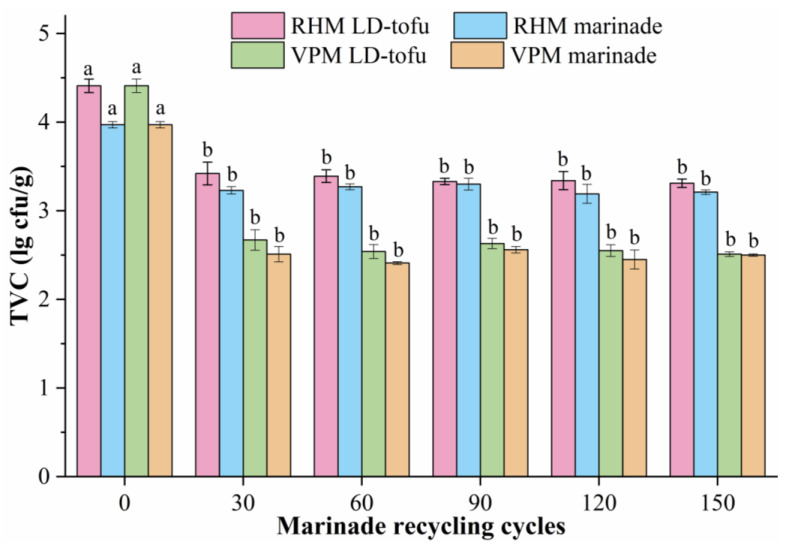
The effects of marinating methods and marinade recycling cycles on the TVC of LD-tofu and marinade. Superscripts with different letters in a row are significantly different between the same sample groups (*p* < 0.05).

The TVC is a core index for monitoring food quality and safety, and is related to the raw materials, processing technology and storage conditions [18,29]. The TVC of the LD-tofu and marinade under different marinating processes and marinade recycling cycles was determined (Figure 3). The initial TVC values of the LD-tofu and marinade were 4.41 and 3.97 lg cfu/g, respectively. With the increase in marinade recycling cycles, the TVC of the LD-tofu and marinade decreased significantly (*p* < 0.05). This indicated that marinating (continuous heating) could effectively reduce the TVC in the product. The higher initial TVC values were caused by the presence of a higher number of microorganisms in the raw materials of LD-tofu and marinade than after marination. This was mutually verified with the findings of Jeong et al. [9]. Numerous studies have shown that spices and herbs (e.g., cinnamon, ginger, bay leaf, cumin, anise) have antibacterial effects [30,31,32]. The marinade used in this study contained more than 20 spices and herbs, and the repeated heating process promoted the release of active substances from them, thereby inhibiting the growth of microorganisms.

As the LD-tofu was subjected to drying and cooling processes after marination, it readily introduced additional microorganisms and hence, the TVC was slightly higher than that of the marinade. With the increase in marinade recycling times, VPM LD-tofu’s TVC decreased from 4.41 lg cfu/g to 2.51–2.67 lg cfu/g as a result of the marinating process, which had a significant inhibitory effect. The TVC of the VPM LD-tofu and VPM marinade was significantly lower than that of RHM LD-tofu and RHM marinade. This indicated that the quality and safety of the VPM LD-tofu and VPM marinade were higher, meaning that conducting the marinating process in a confined space could play a key role in the TVC of the LD-tofu and marinade.

### 3.4. Changes in Bacterial α-Diversity and β-Diversity of LD-tofu and Marinade under Different Marinating Processes and Different Marinade Recycling Cycles

Bacterial α-diversity analysis can reflect the diversity, evenness and richness of bacterial communities in a sample [33]. The Shannon, Simpson, Chao1 and ACE indexes of bacterial α-diversity, effective tags and operational taxonomic units (OTUs) in the LD-tofu and marinade were analysed in this study (Table 2). With the increase in marinade recycling cycles, the effective tags in the LD-tofu and marinade decreased significantly, which was consistent with the trend in TVC. With the increase in marinade recycling time, the number of OTUs in the LD-tofu decreased slightly and then significantly increased, while the number of OTUs in the marinade significantly increased (*p* < 0.05). This showed that the marinating process reduced the number of bacterial communities in the LD-tofu and marinade but increased the diversity of bacterial community species.

The Simpson and Shannon indexes can comprehensively reflect the evenness and richness of sample species. The larger these values, the greater the richness and evenness [33]. The Chao1 and ACE indexes mainly reflect the diversity information of sample species. The higher these values, the greater the number of species [34]. With the increase in marinade recycling cycles, the Shannon, Simpson, Chao1 and ACE indexes of bacterial communities in the LD-tofu and marinade showed different increasing trends, which was consistent with the trend in the number of OTUs. These results indicated that the marinating process could improve the diversity and richness of bacterial communities in the LD-tofu and marinade.

**Figure 4:**
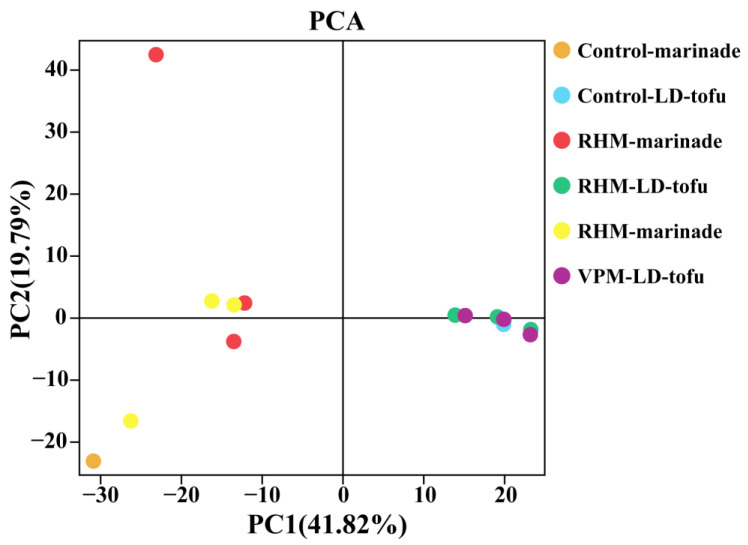
Principal component analysis of the effects of marinating methods and marinade recycling cycles on the microbial β-diversity of LD-tofu and marinade.

Principal component analysis (PCA) is a common method for β-diversity analysis. It can describe the similarity–difference relationships of bacterial communities between different samples [35]. In the LD-tofu and marinade, PC1 and PC2 contributed 41.82% and 19.79%, respectively. This means that PC1 and PC2 explained 61.61% of the difference between groups (Figure 4). The greater the distance between sample points in the PCA, the greater the bacterial community difference between them. In contrast, the closer the sample points, the more similar the bacterial community composition between the two samples [35]. With the increase in the marinade recycling cycles, the marinade points separated. This indicated that there were significant differences between marinade samples and that the bacterial community was in a state of dynamic change during the marinating process. However, all the LD-tofu points overlapped, indicating that the difference between LD-tofu samples was not significant and the similarity of bacterial communities was high.

According to the α-diversity and β-diversity analysis results, the initial bacterial diversity in the LD-tofu was higher than that in the marinade. The reason could be attributed to the fact that LD-tofu is a reprocessed tofu product, and tofu becomes rich in beneficial microorganisms after being curdled by fermented soybean whey (mixed fermentation with multiple strains) [17]. Additionally, the diversity and richness of microbial communities in the VPM LD-tofu and VPM marinade were significantly higher than that in the RHM LD-tofu and RHM marinade. However, the TVC in the VPM LD-tofu and VPM marinade was significantly lower than that in the RHM LD-tofu and RHM marinade. This indicated that the VPM could not only reduce the TVC more effectively but also protect the structural balance of microbial communities.

### 3.5. Changes in Bacterial Community Composition of LD-tofu and Marinade under Different Marinating Processes and Different Marinade Recycling Cycles

**Figure 5:**
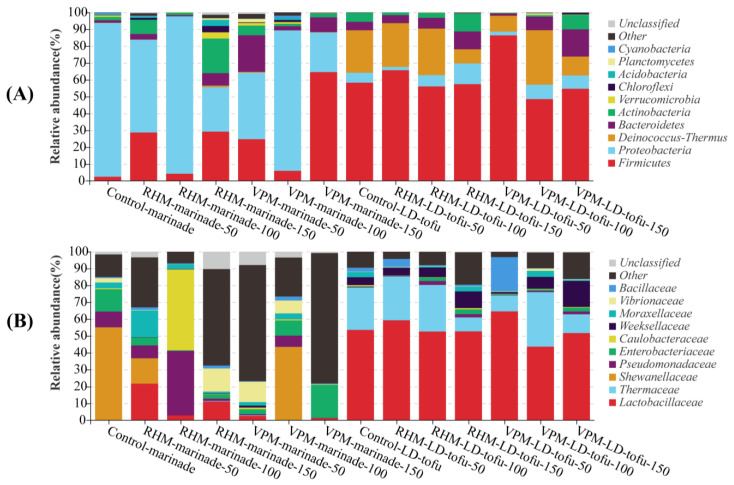
Effects of marinating methods and marinade recycling cycles at the (**A**) phylum and (**B**) family bacterial community level in LD-tofu and marinade.

In this study, 16S rDNA was extracted from the LD-tofu and marinade for PCR amplification and sequencing. The changes in the bacterial community composition of the LD-tofu and marinade under different marinating processes and different marinade recycling cycles were determined by database comparison (Figure 5). At the phylum level, *Proteobacteria*, *Firmicutes* and *Actinobacteria* were the predominant bacteria in all samples, accounting for 81.20–94.77% (Figure 5A). The top 10 bacteria at the phylum level also included *Deinococcus-Thermus*, *Bacteroidetes*, *Verrucomicrobia*, *Chloroflexi* and *Planctomycetes*. The *Proteobacteria* and *Firmicutes* in the marinade accounted for 80.50–93.66%. The higher abundance of *Proteobacteria* and *Firmicutes* in the marinade could be due to the spices and herbs as they are often present in plant soil [36,37]. The *Firmicutes* and *Deinococus-Thermus* in the LD-tofu accounted for 80.35–83.47%. The presence of *Firmicutes* in the control LD-tofu could be due to soybean picking or tofu residue. The increase in the *Firmicutes* in the LD-tofu after the marinating process might be due to the marinade’s high *Firmicutes* content [19,38]. The predominant phylum-level bacteria of the LD-tofu or marinade exhibited a high degree of similarity under different marinating processes and marinade recycling cycles. However, there were significant differences between the dominant bacteria of the LD-tofu and marinade. Additionally, the relative abundance of each dominant bacteria differed significantly.

At the family level, *Lactobacillaceae* were the predominant bacteria in the LD-tofu (Figure 5B). It was inevitable that the tofu and its reprocessed products would contain considerable *Lactobacillus* because the tofu was curdled by *Lactobacillus*-fermented soybean whey [17]. Additionally, *Lactobacillaceae* are widely found in plants, equipment surfaces and water. They can also be introduced into products during processing [39,40]. The abundance of *Lactobacillaceae* decreased with the increase in marinade recycling cycles. It was 65.23%, 48.13% and 44.32% in the control LD-tofu, VPM LD-tofu and RHM LD-tofu, respectively. These results indicated that the marinating process had a greater intervention effect on *Lactobacillaceae*. The *Shewanellaceae* in the control marinade accounted for 54.92%. *Shewanellaceae* are extremely adaptable to the environment and can survive and reproduce under different salt concentrations, temperatures and other environments [41]. The identification of *Shewanellaceae* in this work provides a theoretical basis for the sterilisation and safety control of LD-tofu. With the increase in marinade recycling cycles, *Lactobacillaceae*, *Pseudomonaceae*, *Caulobacteraceae* and *Moraxellaceae* in the RHM marinade replaced *Shewanellaceae* as the predominant bacteria, whereas *Enterobacteriaceae*, *Hewanellaceae* and *Vibrionaceae* became the predominant bacteria in the VPM marinade.

**Figure 6:**
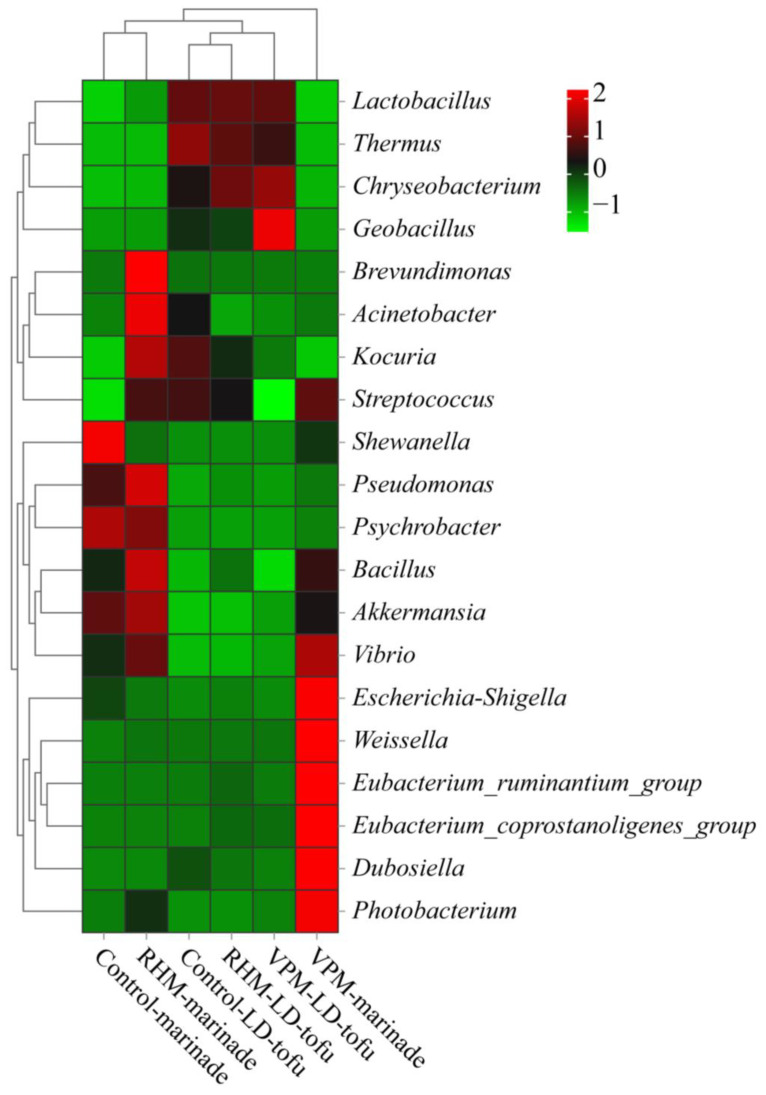
Effects of marinating methods and marinade recycling cycles at the bacterial community genus level in LD-tofu and marinade.

To comprehensively analyse the bacterial community composition in the LD-tofu and marinade, genus-level bacteria with a relative abundance of greater than 0.1% were selected for cluster analysis (Figure 6). The relative abundance of genus-level bacteria in the marinade of the control, VPM and RHM groups changed most dynamically, which was similar to the results of Correa-Galeote et al. [40]. *Shewanella* and *Psychrobacter* were the predominant bacteria in the control marinade, while *Brevundimonas*, *Acinetobacter*, *Kocuria*, *Pseudomonas*, *Bacillus* and *Akkermania* were the predominant bacteria in the RHM marinade. *Escherichia-Shigella*, *Weissella*, *Eubacterium_ruminantium_group*, *Eubacterium_coprostanoligenes_group*, *Dubosiella* and *Photobacterium* were the predominant bacteria in the VPM marinade. There might be several reasons for the significant difference in the composition of dominant bacteria in the marinade samples. First, the marinade is rich in nutrients and high in water activity, which can promote bacterial growth and reproduction [42]. Second, a certain proportion of control marinade should be added before a new marinating process. Therefore, the control marinade can readily breed bacteria when it is not stored properly. Third, the predominant microorganisms in the LD-tofu could move into the marinade during the marinating process. Fourth, the differences in the marinating process parameters, such as temperature and open vs. closed environment, could have a significant impact on the microorganisms [42,43].

The relative abundance of genus-level bacteria in the control, VPM group and RHM group LD-tofu were highly similar. Since *Lactobacillus* was the crucial bacterium for fermented soybean whey (tofu coagulant) preparation, the relative abundance of Lactobacillus in the LD-tofu was relatively high and did not change significantly [17,44]. Compared with the control LD-tofu group, the relative abundance of *Thermus* in the RHM LD-tofu and VPM LD-tofu groups decreased slightly, while the relative abundance of *Streptococcus* decreased significantly. These phenomena occurred because the RHM and VPM were performed at 80 °C and 90 °C, respectively, both of which were much higher than the optimal growth temperature of *Streptococcus* (37 °C) and *Thermus* (70–72 °C) [45,46]. The relative abundance of *Chryseobacterium* and *Geobacillus* in the RHM LD-tofu and VPM LD-tofu was higher than that in the control LD-tofu. These results showed that the bacterial community of the LD-tofu samples was similar under different marinating processes. However, there were significant differences in the content of specific bacteria at the genus level.

### 3.6. Correlation Analysis of Bacterial Community and Quality Characteristics of LD-tofu and Marinade

**Figure 7:**
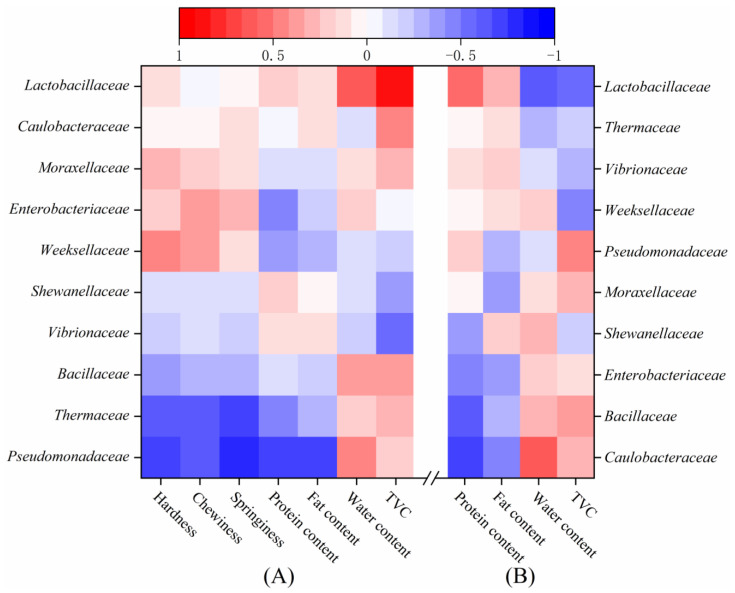
Pearson correlation between the bacterial community family and the quality characteristics of (**A**) LD-tofu and (**B**) marinade.

Pearson’s correlation test was used to determine the correlation between the family-level bacterial community and the quality characteristics of the LD-tofu and marinade (Figure 7). *Pseudomonadaceae* and *Thermaceae* in the LD-tofu were negatively correlated with the hardness, chewiness, springiness, protein content and fat content, but positively correlated with the water content and TVC (Figure 7A). This could be because *Pseudomonadaceae* can produce lipase and protease [47,48], which could reduce the fat content and protein while changing the textural characteristics. Previous studies have confirmed that equalisin (the thermo-active serine peptidase of *Thermous aquaticus*) can deteriorate the texture of food [49], which was verified by the results of this study. The presence of *Lactobacillacea* in LD-tofu was positively correlated with the water content and TVC. This might be because the *Lactobacillacea* in the LD-tofu gradually infiltrated the marinade during the marinating process, which was consistent with the change in water content and TVC in LD-tofu.

*Caulobacteriaceae*, *Bacillaceae* and *Enterobacteriaceae* in the marinade were negatively correlated with the protein and fat content but positively correlated with the water content and TVC (Figure 7B). *Caulobacteriaceae*, *Bacillaceae* and *Enterobacteriaceae* are often found in raw materials (including spices, herbs and drinking water) [42,50,51]. Although heating can effectively control these bacteria, a certain proportion of control marinade was added before commencing a new marinating process. This resulted in a high content of these bacteria in the marinade. *Lactobacillaceae* in the marinade was negatively correlated with the water content and TVC. This might be because *Lactobacillaceae* in the LD-tofu infiltrated the marinade during the marinating process. Its change trend was opposite to the change in the water content and TVC in the marinade.

## 4. Conclusions

In this study, the effects of different marinating processes on the nutritional components, textural characteristics, TVC and bacterial community of LD-tofu were analysed. The marinating process promoted the loss of nutrients in LD-tofu, resulting in a significant increase in the marinade protein and fat content. The textural characteristics of LD-tofu were improved under different marinating processes. The TVC values of the LD-tofu and marinade were significantly reduced following marination. The nutrient content, textural characteristics and microbial contamination of the VPM LD-tofu were superior to those of the RHM LD-tofu. There were also significant differences in the bacterial communities of the LD-tofu and marinade under different marinating processes. *Pseudomonadaceae*, *Thermaceae* and *Lactobacillaceae* were closely related to the quality characteristics of LD-tofu, while *Caulobacteriaceae*, *Bacillaceae* and *Enterobacteriae* were closely related to the quality characteristics of the marinade. The present work provides producers with a solid theoretical basis to maintain the quality and safety of LD-tofu.

## Figures and Tables

**Table 1:** Effects of different marinating methods and marinade recycling cycles on the textural characteristics of LD-tofu.

Textural Properties	Groups	Marinade Recycling Cycles					
		0	30	60	90	120	150
Chewiness/g	RHM	375.19 ± 2.65 ^a^	352.08 ± 2.45 ^b^	360.92 ± 0.57 ^c^	373.15 ± 0.47 ^ad^	380.65 ± 7.44 ^e^	387.28 ± 1.42 ^f^
	VPM		365.42 ± 4.34 ^b^ *	373.32 ± 0.46 ^ac^ *	398.65 ± 1.95 ^d^ *	415.47 ± 2.13 ^e^ *	429.22 ± 8.32 ^f^ *
Hardness/g	RHM	581.78 ± 3.67 ^a^	542.64 ± 5.01 ^b^	554.95 ± 0.55 ^c^	574.59 ± 4.15 ^d^	592.29 ± 2.61 ^e^	597.12 ± 0.28 ^f^
	VPM		551.90 ± 4.79 ^b^ *	559.41 ± 0.80 ^c^ *	573.09 ± 0.85 ^d^	608.79 ± 5.88 ^e^ *	620.32 ± 4.38 ^e^ *
Springiness	RHM	0.885 ± 0.051 ^a^	0.877 ± 0.017 ^a^	0.880 ± 0.003 ^a^	0.884 ± 0.035 ^ac^	0.896 ± 0.011 ^d^	0.903 ± 0.008 ^d^
	VPM		0.879 ± 0.040 ^a^	0.883 ± 0.007 ^a^	0.903 ± 0.028 ^b^ *	0.915 ± 0.005 ^c^ *	0.927 ± 0.011 ^d^*

Note: Superscripts with different letters in a row are significantly different between groups (*p* < 0.05). * indicates a significant difference between RHM groups and VPM groups (*p* < 0.05).

**Table 2:** The effects of marinating methods and marinade recycling cycles on the microbial α-diversity of LD-tofu and marinade.

Indexes	Sample	Marinade Recycling Cycles			
		0	50	100	150
Effective Tags	RHM-LD-tofu	114,911 ± 142.37 ^a^	109,489 ± 171.74 ^b^	100,387 ± 301.38 ^c^	101,420 ± 212.42 ^c^
	VPM-LD-tofu		106,241 ± 80.96 ^b^	93,938 ± 128.07 ^c^	82,335 ± 197.81 ^d^
	RHM-marinade	98,858 ± 62.09 ^a^	91,009 ± 237.16 ^b^	90,038 ± 92.49 ^b^	85,578 ± 82.30 ^c^
	VPM-marinade		85,551 ± 102.34 ^b^	81,317 ± 157.90 ^c^	72,668 ± 42.51 ^d^
OTUs	RHM-LD-tofu	330 ± 2.15 ^a^	295 ± 1.03 ^b^	368 ± 2.46 ^c^	394 ± 1.61 ^d^
	VPM-LD-tofu		297 ± 0.67 ^b^	371 ± 1.08 ^c^	410 ± 1.47 ^d^
	RHM-marinade	263 ± 0.85 ^a^	279 ± 1.82 ^a^	307 ± 0.91 ^b^	363 ± 0.47 ^c^
	VPM-marinade		310 ± 0.43 ^b^	346 ± 1.52 ^c^	397 ± 0.29 ^d^
Shannon	RHM-LD-tofu	4.46 ± 0.07 ^a^	5.04 ± 0.09 ^b^	5.47 ± 0.07 ^c^	5.27 ± 0.05 ^d^
	VPM-LD-tofu		5.69 ± 0.01 ^b^	5.56 ± 0.06 ^c^	5.76 ± 0.09 ^d^
	RHM-marinade	4.20 ± 0.05 ^a^	4.64 ± 0.02 ^b^	4.80 ± 0.04 ^b^	5.64 ± 0.07 ^c^
	VPM-marinade		4.71 ± 0.03 ^b^	4.95 ± 0.01 ^c^	5.70 ± 0.06 ^d^
Simpson	RHM-LD-tofu	0.755 ± 0.011 ^a^	0.802 ± 0.009 ^b^	0.825 ± 0.017 ^c^	0.821 ± 0.008 ^c^
	VPM-LD-tofu		0.782 ± 0.006 ^b^	0.848 ± 0.022 ^c^	0.805 ± 0.003 ^d^
	RHM-marinade	0.715 ± 0.005 ^a^	0.760 ± 0.000 ^b^	0.747 ± 0.009 ^c^	0.774 ± 0.008 ^d^
	VPM-marinade		0.783 ± 0.002 ^b^	0.796 ± 0.007 ^c^	0.811 ± 0.013 ^c^
Chao1	RHM-LD-tofu	437.05 ± 0.60 ^a^	390.00 ± 2.67 ^b^	459.13 ± 1.33 ^c^	449.93 ± 1.28 ^c^
	VPM-LD-tofu		377.94 ± 1.92 ^b^	492.35 ± 2.11 ^c^	513.88 ± 0.89 ^d^
	RHM-marinade	289.06 ± 1.03 ^a^	337.57 ± 2.01 ^b^	416.05 ± 1.08 ^c^	462.40 ± 0.43 ^d^
	VPM-marinade		402.34 ± 0.87 ^b^	455.06 ± 0.94 ^c^	499.69 ± 1.57 ^d^
Ace	RHM-LD-tofu	390.43 ± 1.14 ^a^	357.28 ± 2.31 ^b^	479.47 ± 0.61 ^c^	459.29 ± 1.42 ^d^
	VPM-LD-tofu		361.53 ± 1.09 ^b^	506.41 ± 1.86 ^c^	507.54 ± 2.52 ^c^
	RHM-marinade	290.63 ± 0.97 ^a^	322.67 ± 0.74 ^b^	462.01 ± 2.49 ^c^	474.73 ± 1.66 ^d^
	VPM-marinade		434.37 ± 1.40 ^b^	557.89 ± 1.91 ^c^	600.25 ± 0.39 ^d^

Note: Superscripts with different letters in a row are significantly different between groups (*p* < 0.05).

## Data Availability

Data are contained within the article.
